# Signature of coexistence of superconductivity and ferromagnetism in two-dimensional NbSe_2_ triggered by surface molecular adsorption

**DOI:** 10.1038/ncomms11210

**Published:** 2016-04-04

**Authors:** Xiaojiao Zhu, Yuqiao Guo, Hao Cheng, Jun Dai, Xingda An, Jiyin Zhao, Kangzhen Tian, Shiqiang Wei, Xiao Cheng Zeng, Changzheng Wu, Yi Xie

**Affiliations:** 1 Hefei National Laboratory for Physical Sciences at the Microscale, iChEM (Collaborative Innovation Center of Chemistry for Energy Materials), CAS Center for Excellence in Nanoscience, and CAS Key Laboratory of Mechanical Behavior and Design of Materials, University of Science and Technology of China, Hefei 230026, China; 2 National Synchrotron Radiation Laboratory, University of Science and Technology of China, Hefei, Anhui 230029, China; 3 Department of Chemistry, University of Nebraska-Lincoln, Lincoln, Nebraska 68588, USA

## Abstract

Ferromagnetism is usually deemed incompatible with superconductivity. Consequently, the coexistence of superconductivity and ferromagnetism is usually observed only in elegantly designed multi-ingredient structures in which the two competing electronic states originate from separate structural components. Here we report the use of surface molecular adsorption to induce ferromagnetism in two-dimensional superconducting NbSe_2_, representing the freestanding case of the coexistence of superconductivity and ferromagnetism in one two-dimensional nanomaterial. Surface-structural modulation of the ultrathin superconducting NbSe_2_ by polar reductive hydrazine molecules triggers a slight elongation of the covalent Nb–Se bond, which weakens the covalent interaction and enhances the ionicity of the tetravalent Nb with unpaired electrons, yielding ferromagnetic ordering. The induced ferromagnetic momentum couples with conduction electrons generating unique correlated effects of intrinsic negative magnetoresistance and the Kondo effect. We anticipate that the surface molecular adsorption will be a powerful tool to regulate spin ordering in the two-dimensional paradigm.

## Abstract (web summary)

Ferromagnetism and superconductivity possess inherently incompatible electronic spin ordering, and their coexistence requires elaborate engineering of material components. Here, the authors induce ferromagnetism in a two-dimensional superconducting crystal by the adsorption of hydrazine molecules.

## Graphical abstract

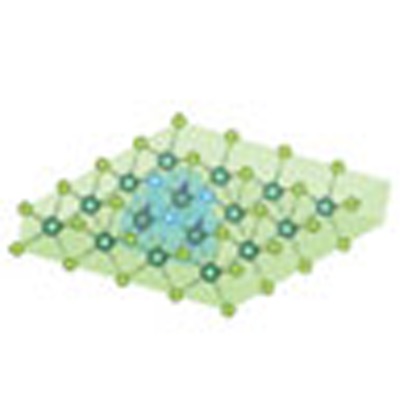

Since the discovery of superconductivity (SC), the interplay between SC and magnetism has been a topic of cutting-edge research in solid-state materials^1^,^2^,^3^,^4^,^5^,^6^,^7^,^8^. Given that ferromagnetism (FM) often plays a destructive role in superconductors by damaging the singlet correlations responsible for the pairing interaction^9^,^10^,^11^, the coexistence of SC and FM is rare yet valuable. The well-established correlated systems that exhibit these two competing electronic states are usually prepared by fabricating two separate structures, taking advantage of the inherent properties of each individual parent ingredient. For instance, such systems include the hybrid [Ni_0.66_Al_0.33_(OH)_2_][TaS_2_] system, which is composed of ferromagnetic cation layers and superconducting anion layers^12^; Fe/Nb/Fe trilayers consisting of one single SC layer (Nb layer) between two FM layers (Fe layer)^13^; and the layered [(Li_1−*x*_Fe_*x*_)OH](Fe_1−*y*_Li_*y*_)Se system, which consists of ferromagnetic (Li_1−*x*_Fe_*x*_)OH and superconducting (Fe_1−*y*_Li_*y*_)Se layers^14^. These fascinating findings achieved by virtue of the proximity effect have generated increased interest in investigations of the interplay between SC and FM and have provided insight into the underlying physics of SC. However, further realization of the coexistence of these two competing electronic states in a single homogeneous structure promises further insight into the coupling of these two competing orderings. In this regard, the recently reported two-dimensional (2D) electron liquid LaAlO_3_/SrTiO_3_ interface^15^,^16^,^17^ represents an advance in the coexistence of SC and FM, as both ingredients are neither superconducting nor ferromagnetic in their individual states. The coexistence of these properties at a uniform interface makes this 2D electronic system an attractive platform for achieving the coexistence of SC and FM in one system. The question remains whether the coexistence of both electronic states can be designed in a single freestanding 2D electronic structure, with the expectation of unconventional SC in the ground state for the stronger interplay of FM and SC.

Recently, two-dimensional or interface superconductors with the thickness down to one unit cell or atomic layers have attracted much attention and demonstrated many intriguing behaviours^18^,^19^,^20^. 2D transitional metal chalcogenides, as the classic prototype for superconductors^21^,^22^,^23^,^24^,^25^,^26^,^27^, provide a promising material platform for investigation of the coexistence of SC and FM because of their unique spin configuration and richly correlated electronic phases. 2H-NbSe_2_, a canonical layered SC material, consists of one Nb layer sandwiched between two Se layers, forming trigonal prismatic NbSe_6_ with covalent in-plane bonds; this material exhibits the highest superconducting transition ever reported in the layered transitional metal chalcogenide family (7.4 K; refs 28, 29). The transition metal element Nb^4+^, in the [Kr]4*d*^1^ configuration, is particularly *d* characterized and is thus expected to demonstrate a range of magnetic response. Nevertheless, pristine NbSe_2_ is nonmagnetic because the Nb atoms are hybridized with the Se atoms to form a covalent Nb–Se interaction, which quenches the magnetic moment of the Nb ions. Although there remains no effective way to induce FM in this system, let alone the coexistence of SC and FM, theoretical calculations^30^,^31^ predict that the suppressed magnetism can be expressed by modifying the covalent interaction between the Nb and Se atoms under applied strain, which can result in more unpaired electrons around the Nb atoms, theoretically resulting in macro-magnetic momentum. Moreover, the adjacent Se–Nb–Se layers are coupled to each other by weak van der Waals interactions, rendering it feasible to achieve a free-standing ultrathin structure. Of note, the ultrathin structure characteristics leave much space for the chemical design to obtain surface-structural modulation, which depends on the chemical versatility of the surface layer^32^,^33^. In this regard, ultrathin NbSe_2_ nanosheets would be a new class of 2D electronic systems that possess coupled SC and FM.

Here we highlight a surface-structural modulation strategy for the incorporation of intrinsic FM into the superconducting NbSe_2_ framework, thereby accomplishing the structural integration of SC and FM in a freestanding case of 2D nanomaterial. The surface adsorption of polar reductive hydrazine molecules triggers structural modulation while the pristine structure is reserved, leading to the elongated covalent Nb–Se bonds and the impairment of the Nb–Se covalent interactions, which enhance the ionicity of the tetravalent Nb with unpaired electrons and thus successfully yields ferromagnetic ordering in superconducting 2D NbSe_2_ nanomaterials. The coexistence of SC and FM in a single 2D nanomaterial gives rise to the fascinating coupled effects of negative magnetoresistance (MR) and the Kondo effect. This work provides a route for achieving the integration of ordered magnetism and SC in 2D systems.

## Results

### Surface molecular adsorption

**Figure 1:**
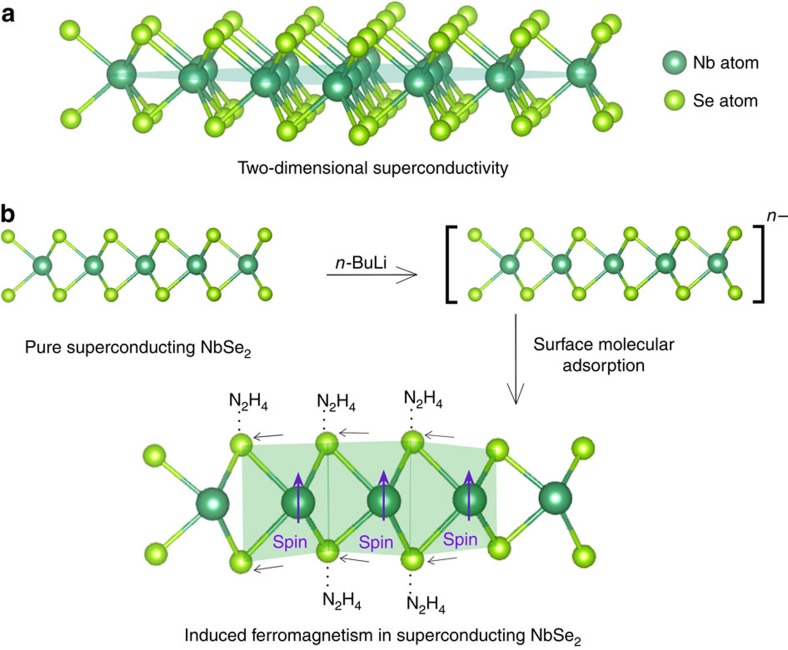
Schematic illustration of the coexistence of superconductivity and ferromagnetism in two-dimensional NbSe_2_. (**a**) Side view of the two-dimensional superconducting NbSe_2_. (**b**) The Li-intercalated exfoliated two-dimensional NbSe_2_ is negatively charged, and *n*^−^ indicated the excess charges carried by the nanosheets. By surface molecular adsorption, the electrostatic interactions (marked in dashed line) of the negative-charge nanosheets with polar hydrazine molecules triggered the surface-structural distortion of Nb–Se bond and resulted in the ferromagnetic spin in the superconducting NbSe_2_.

**Figure 2:**
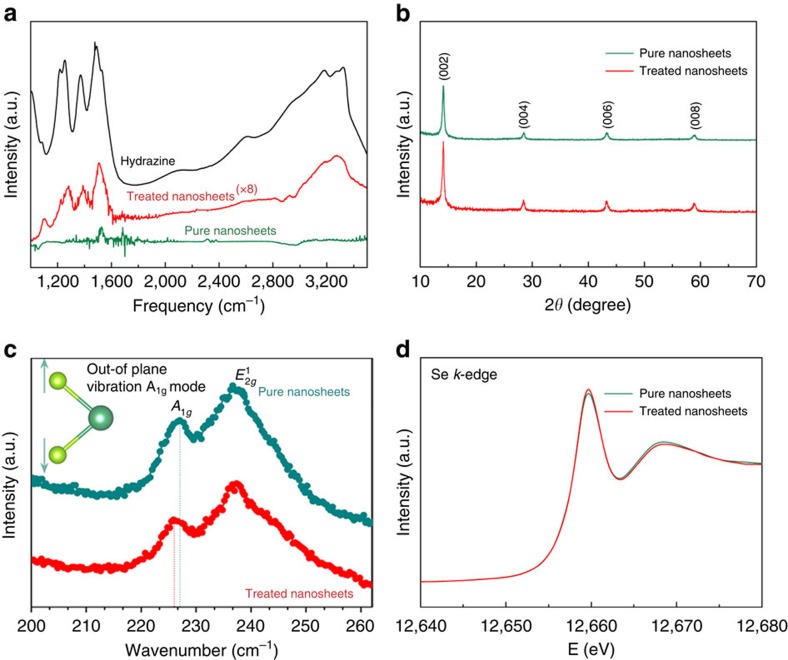
Two-dimensional NbSe_2_ treated by polar hydrazine. (**a**) ATR**-FTIR spectra, revealing the interfacial interaction between the hydrazine molecules and the ultrathin NbSe_2_ nanosheet. (**b**) X-ray diffraction patterns and (**c**) high-resolution Raman spectra of the pure NbSe_2_ and hydrazine-treated NbSe_2_ nanosheets, revealing the retention of long-range order after the treatment. (**d**) Se *K*-edge XANES spectra for the pure NbSe_2_ and hydrazine-treated NbSe_2_ nanosheets, demonstrating the lack of effect on the chemical environment after the treatment.

To enable effective adsorption of polar molecules on the negatively charged NbSe_2_ surface^34^, the assembled thin film of NbSe_2_ ultrathin nanosheets (Supplementary Figs 1 and 2, Supplementary Notes 1 and 2) was exposed in the vapour flow of hydrazine at an elevated temperature of 70 °C, during which the gas state of hydrazine as well as elevated temperature help to overcome the adsorption energy barrier and ensure sufficient interactions between polar hydrazine with nanosheets. In our case, the strong reductive atmosphere plays a vital role in protecting inherent Se–Nb–Se lattice structure from being oxidated, while experimental results have confirmed that other polar molecules such as H_2_O, amines (NH_3_ and so on) and alcohols (CH_3_OH, CH_3_CH_2_OH, and so on) having the relative lower reductive capability inevitably lead to structural destruction with elemental Se particles as by-products. Of note, the assembled thin film of NbSe_2_ ultrathin nanosheets provides abundant interstitial sites to confine and stabilize the absorbed hydrazine molecules (details see Supplementary Fig. 3), ensuring effective surface molecular adsorption to induce local distortions. The surface molecular adsorption of strong reductive hydrazine successfully realized the surface-structural modulation of 2D NbSe_2_ nanosheets as well as the preservation of the NbSe_2_ framework, as schematically illustrated in Fig. 1, where the adsorption site and the resultant structural distortion were highlighted in the green background. The strong polarity of 1.83 Debye of the reductive hydrazine molecules facilitated the surface molecular adsorption due to the electrostatic interactions between the surfaces of the negatively charged NbSe_2_ nanosheets and the adsorbed polar hydrazine molecules, which enabled the attraction of the polar hydrazine molecules to the surfaces of the NbSe_2_ nanosheets, as revealed by the attenuated total reflectance Fourier transform infrared (ATR-FTIR) spectra. The spectrum of the pure hydrazine molecules is presented in the topmost part of Fig. 2a, where the characteristic N–H stretching bond at ∼3,000 cm^−1^ is the signature of the existence of the hydrazine molecules. As expected, the spectrum of the hydrazine-treated NbSe_2_ nanosheets resembles that of the pure hydrazine molecules, thus confirming the adsorption of the hydrazine molecules to the NbSe_2_ nanosheets. By contrast, the spectrum of the pure NbSe_2_ nanosheets does not exhibit a signature similar to that present in the topmost spectrum; in particular, the N–H stretching bond is absent. The hydrazine treatment enabled the adsorption of the hydrazine molecules on the surfaces of the NbSe_2_ nanosheets, thereby triggering local surface-structural distortions.

### Maintenance of pristine NbSe_2_ framework after the adsorption

The pristine Se–Nb–Se layered framework was maintained after surface adsorption of the polar hydrazine molecules, as confirmed by systematic structural characterization performed via X-ray powder diffraction, Raman spectroscopy and Se *K*-edge X-ray absorption near-edge structure (XANES) spectroscopy of the hydrazine-treated NbSe_2_ nanosheets. As observed in Fig. 2b, the X-ray diffraction pattern of the hydrazine-treated nanosheets parallels that of the pure nanosheets, thereby confirming the retention of the NbSe_2_ lattice framework. The high-resolution (0.35 cm^−1^) Raman spectra further confirm that the in-plane structural framework of 2H-NbSe_2_ was well preserved. As observed in Fig. 2c, the Raman spectra of the pure and hydrazine-treated NbSe_2_ nanosheets exhibit two prominent peaks corresponding to the *A*_1*g*_ and 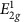
modes of 2H-NbSe_2_ (ref. 35). The slight energy shift (0.9 cm^−1^) of hydrazine-treated NbSe_2_ nanosheets to the lower energy revealed the releasing energy of the out-of-plane vibration *A*_1*g*_ mode, indicating local elongation of Nb–Se covalent bonds that is consistent with X-ray absorption fine structure (XAFS) measurement results in the following discussions. In addition, the Se *K*-edge XANES spectra (Fig. 2d) of the pure and hydrazine-treated NbSe_2_ nanosheets confirm the nearly invisible effect of the hydrazine treatment on the valence states of the element, indicating that there is no electron transfer between the surface-adsorbed hydrazine molecules and the outmost Se layers. In a word, the structural comparison between the pure and hydrazine-treated NbSe_2_ nanosheets revealed that the 2H-NbSe_2_ lattice framework was well preserved at the macroscopic level after the hydrazine treatment.

### Local distortions induced by the adsorption

**Figure 3:**
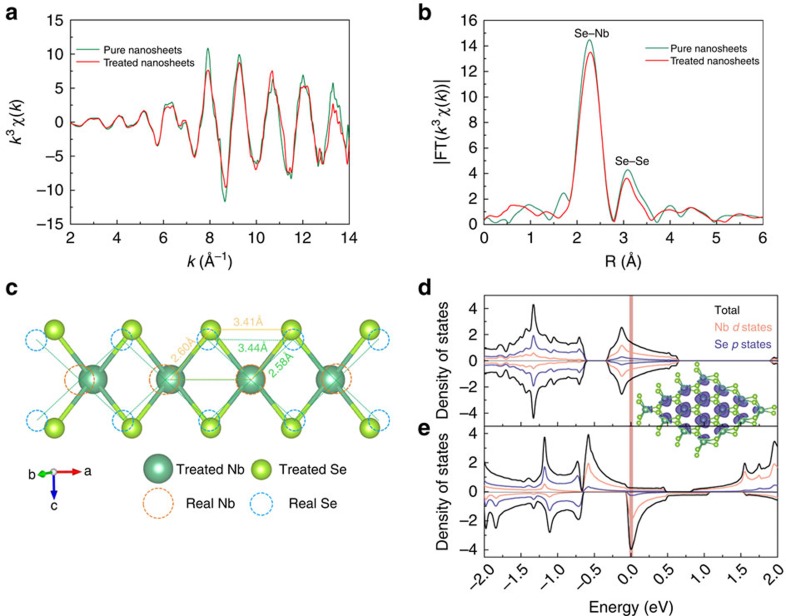
Surface-structural distortion induced by hydrazine molecular adsorption. (**a**) Se *K*-edge extended XAFS oscillation function *k*^3^χ(*k*) and (**b**) the corresponding Fourier transforms FT(*k*^3^χ(*k*)), where *k*=wave vector and *χ*(*k*)=oscillation as a function of the photoelectron wavenumber; (**c**) two-dimensional structural models showing the surface distortion; electronic DOSs of the pure (**d**) and distorted NbSe_2_ nanosheets (**e**) where the inset shows an isosurface plot of the spin density of the distorted NbSe_2_ (isosurface value=0.0053 *e* (a.u.)^−3^).

To examine the local structural modulation after the hydrazine treatment, XAFS measurements were performed to determine the local atomic and electronic structures of the hydrazine-treated NbSe_2_ nanosheets. The *k*^3^χ(*k*) oscillation curve presented in Fig. 3a for the hydrazine-treated NbSe_2_ nanosheets exhibits a spectral shape similar to that of the pure nanosheets; however, a noticeable reduction in the oscillation amplitude is observed. Such an amplitude reduction is also evident in the corresponding Fourier transform (FT) functions. In Fig. 3b, the FT curve of the pure NbSe_2_ nanosheets displays two peaks at ∼2.26 and 3.09 Å, which can be attributed to Se–Nb and Se–Se correlations, respectively. For the hydrazine-treated NbSe_2_ nanosheets, the intensities of these two peaks are significantly decreased, accompanied by a noticeable shift in the position of the Se–Nb peak towards the high-R side and a shift of the Se–Se peak in the opposite direction. The high-quality extended XAFS spectra enabled us to perform further least-squares fittings of the Se *K*-edge data, and the quantitative results thus obtained are summarized in Supplementary Fig. 4 and Supplementary Table 1. Evidently, compared with the pure NbSe_2_ nanosheets, the interatomic distance of the Se–Nb coordination in the hydrazine-treated NbSe2 nanosheets was elongated by 0.02 Å, whereas that of the Se–Se coordination was shortened by 0.03 Å. The resulting remarkable increase in the degree of disorder may have resulted in the damping of both peaks. Notably, in our fitting process, the Se–Se coordination referred to the Se atoms in the *a*–*b* plane, whose coordination number was much greater than that of other types of Se–Se coordination and contributed greatly to the extended X-ray absorption fine structure (EXAFS) signal. Moreover, the negligible hydrazine content was out of consideration in the fitting process. On the basis of these structural parameters, the structural models for hydrazine-treated NbSe_2_ nanosheets are displayed in Fig. 3c, which clearly illustrates the distorted bond lengths and bond angles of the Nb–Se, Nb–Nb and Nb–Se–Nb coordination geometries. It is apparent that, compared with the pure NbSe_2_ nanosheets, the Nb–Se bond length in the hydrazine-treated NbSe_2_ nanosheets was relatively elongated, thus impairing the covalent Nb–Se interactions and augmenting the unpaired electrons around the Nb atoms.

### Surface-structural modulation

As indicated by the comprehensive structural characterizations of the pure and hydrazine-treated NbSe_2_ nanosheets, the adsorption of the polar hydrazine molecules resulted in local modulations in the 2H-NbSe_2_ structure. The electrostatic interactions between the surface charge of the nanosheets and the polar hydrazine molecules successfully triggered local structural modulation while preserving the integrity of the pristine structure. In our case, the hydrazine molecules played a vital role in inducing the structural modulation process. On the one hand, the strong reductive property ensured the structural integrity of NbSe_2_ naonsheets during the experimental process. Notably, representative and available polar molecules with oxidative potentials, such as H_2_O, amines (NH_3_ and so on) and alcohols (CH_3_OH, CH_3_CH_2_OH, and so on), can demolish NbSe_2_ nanosheets, producing Se nanoparticles on the NbSe_2_ nanosheets. The Se 3*d* spectra of H_2_O/CH_3_CH_2_OH/NH_3_-treated NbSe_2_ nanosheets consisted of quadruple peaks, suggesting the mixed selenium valence states, especially the additional doublet peak of Se^0^ 3*d*_3/2_ (55.7 eV) and Se^0^ 3*d*_5/2_ (54.9 eV), as shown in Supplementary Fig. 5 and Supplementary Note 3, which in turn confirmed the protective role of reductive atmosphere produced by hydrazine molecules. On the other hand, the N atom in the hydrazine molecule is threefold coordinated with its counterpart N atom and two H atoms, and this threefold coordination, together with the structural asymmetry of the molecule, results in the strong polarity of the hydrazine molecule of 1.83 Debye, which is the strongest polarity among the available reductive vapour molecules and advantageous over other counterparts. The strong polarity boosts the surface molecular adsorption, which is beneficial to the surface-structural modification. Hence, the adsorption of polar reductive hydrazine molecules successfully induced surface-structural modulation via the elongation of the Nb–Se bonds, which impaired the Nb–Se covalent interaction and enhanced the ionicity of the tetravalent Nb with unpaired electrons, endowing the distorted 2D NbSe_2_ with an intriguing magnetic response.

### Polarized spin calculations

Spin-polarization calculations were performed to gain an understanding of the anticipated FM of the surface-distorted NbSe_2_. In the inset of Fig. 3e, the purple isosurface represents the spin density, which is rather spherical around the Nb atoms, thereby revealing the ferromagnetic order incorporated into the distorted NbSe_2_ nanosheets. The densities of states, shown in Fig. 3d,e, provide further evidence for this phenomenon: the significant asymmetry in the density of states (DOS) between the spin-down channel and the spin-up channel provides direct evidence of the intrinsic FM in the distorted NbSe_2_ nanosheets. By contrast, the densities of states for the spin-up and spin-down channels of the pure NbSe_2_ nanosheets are degenerate, answering for the nonmagnetic property of the pure NbSe_2_ nanosheets. From examining the atomic site-projected DOSs, it is apparent that the states near the Fermi level for the distorted NbSe_2_ are primarily contributed by Nb *d* states. The polarized spin calculations suggest that the delicate surface distortions caused by the elongated Nb–Se bond induce ordered spin behaviour.

### Induced FM in the distorted NbSe_2_

**Figure 4:**
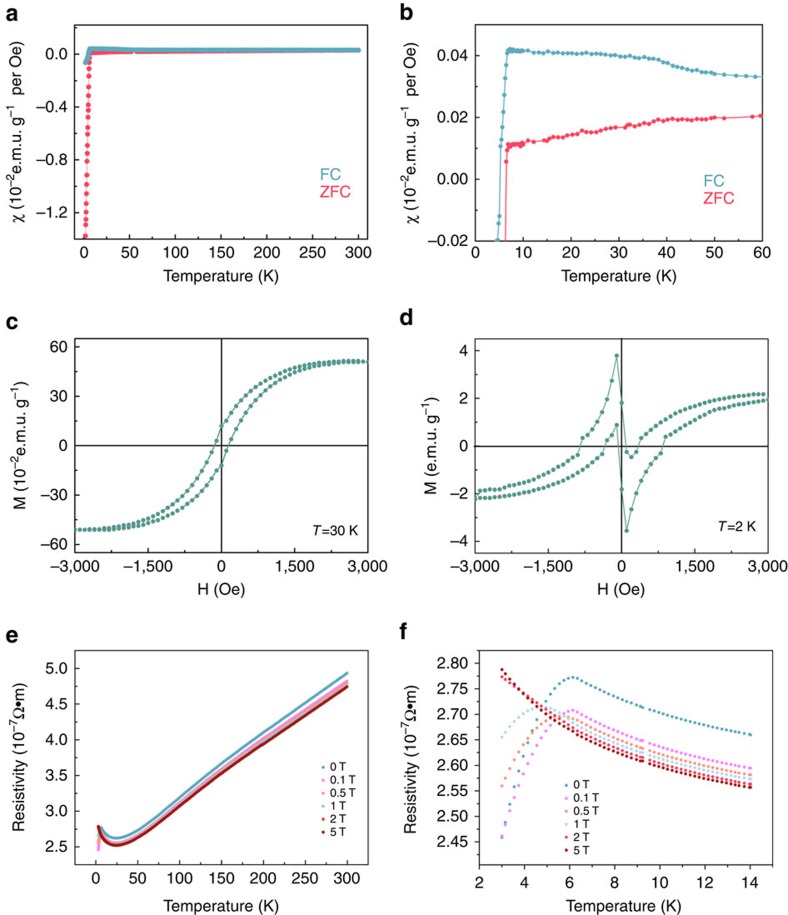
Coexistence of superconductivity and ferromagnetism in hydrazine-treated nanosheets. (**a**) Temperature dependence of the magnetic susceptibility *χ* of the hydrazine-treated NbSe_2_ nanosheets under a magnetic field of 200 Oe for FC and ZFC processes; (**b**) a magnified view of the *χ*–*T* curve at low temperature; (**c**,**d**) isothermal magnetizations (*M*–*H* curves) at 30 and 2 K, respectively; (**e**) temperature dependence of the resistivity (*R*–*T*) under various magnetic fields; (**f**) a magnified view of the *R*–*T* curve at low temperature.

The modulation of the covalent Nb–Se interaction in the hydrazine-treated NbSe_2_ nanosheets induced ordered spin in this SC system, as verified by magnetic measurements. Figure 4a shows the temperature dependence of the magnetic susceptibility (*χ*) from 2 to 300 K for the hydrazine-treated NbSe_2_ nanosheets. These data were collected under a magnetic field of 200 Oe using both zero-field-cooling (ZFC) and field-cooling (FC) processes performed on the assembled film of NbSe_2_ ultrathin nanosheets. Both the ZFC and FC curves of the hydrazine-treated nanosheets exhibit a diamagnetic signal (superconducting transition) at ∼6.8 K, providing evidence for the preservation of SC after treatment. For comparison, the corresponding pure NbSe_2_ sample, the diamagnetic signal appeared at 7.4 K and the behaviour of *χ* in the normal state were weakly temperature-dependent, as observed in Supplementary Fig. 6a. It is clearly evident that after the treatment not only did the superconducting transition temperature decrease but also the behaviour of *χ* in the normal state was more strongly temperature-dependent. There was an obvious transition at ∼40 K, as shown in Fig. 4b, where the susceptibility *χ* of the FC process notably increased below 40 K, thus indicating the onset of FM behaviour. To identify the anticipated FM of the hydrazine-treated SC system, the isothermal magnetization curves (*M*–*H* curves) at 30 and 2 K were measured; these curves are presented in Fig. 4c,d, respectively. The *M*–*H* curve at 30 K shown in Fig. 4c consists of a typical hysteresis loop, which clearly implies the successful introduction of FM into the SC NbSe_2_ system. Furthermore, the isothermal magnetization curve in the superconducting region (2 K) suggests the coexistence of SC with the ferromagnetic ordering. Of note, considering the 2D feature of NbSe_2_ nanosheets and the random configuration of surface molecular adsorption, the induced ferromagnetic state in our case is not the conventional long-range spin ordered states but the short-range magnetic domains^16^,^26^. As observed in Fig. 4d, the *M*–*H* curve at 2 K exhibits an obvious diamagnetic signal in the low-field region, whereas the diamagnetic behaviour eventually transforms into FM with an increasing applied field.

## Discussion

To probe the interplay between the induced FM and the inherited SC, the magnetic response of the transport properties of the 2D NbSe_2_ was investigated, of which the optical image is shown in Supplementary Fig. 2. As observed in Fig. 4e,f, as the temperature was decreased, the resistivity of the treated NbSe_2_ sample slowly decreased, similar to the result obtained for pure NbSe_2_ (see Supplementary Fig. 6d). However, there were three significantly different *R–T* behaviours between the hydrazine-treated and pure samples. First, the value of the resistivity at zero magnetic field for the treated sample was larger than that for the pure sample. Second, the pure sample exhibited an ordinary positive MR, defined as (*ρ*_H_−*ρ*_0_)/*ρ*_0_, where *ρ*_H_ and *ρ*_0_ represent the resistivity under a magnetic field and the primitive resistivity, respectively. This ordinary positive MR commonly exists in metals. By comparison, the hydrazine-treated sample exhibited an abnormal negative MR of −6% in the non-superconducting region. Third, the pure NbSe_2_ sample exhibited normal metal behaviour (that is, positive coefficient of resistance to temperature) until the superconducting transition at 7.4 K, whereas the resistivity of the hydrazine-treated sample initially decreased with decreasing temperature until a minimum was reached at ∼30 K and subsequently increased as the temperature approached the superconducting temperature. The abnormal increase in the resistivity below 30 K can be well fitted to the model of the Kondo effect^36^, revealing a logarithmic temperature dependence for the resistivity, as shown in Supplementary Fig. 7.

We believe that the different *R*-*T* behaviours of the pure NbSe_2_ and hydrazine-treated NbSe_2_ nanosheets can be attributed to the introduction of local magnetic momentum via hydrazine treatment in the metal NbSe_2_ matrix. The increased resistivity of the hydrazine-treated NbSe_2_ compared with that of the pure NbSe_2_ is interpretable when considering the scattering effect between the induced local magnetic momentum and the conduction electrons. Besides, the external magnetic field increased the effective arrangement of the localized spins and thus suppressed the fluctuations of the local spins in space and time, thereby leading to a decrease in the scattering effect between the local magnetic momentum and conduction electrons and explaining the negative MR observed in the hydrazine-treated NbSe_2_ sample. Furthermore, the observation of the Kondo scattering in the FM onset temperature region, where the susceptibility exhibited an obvious increase, also confirms the presence of ferromagnetic scattering centres at low-temperature region in the hydrazine-treated NbSe_2_ nanosheets. Those unique phenomena of the negative MR and the Kondo effect observed in a uniform 2D nanomaterial indicate direct interaction between the conducting electrons and the local magnetic moment, beyond the proximity effect. Therefore, FM and SC were observed simultaneously in the distorted NbSe_2_ nanosheets as a result of surface-structural modulation, based on the electronic phase separation model, where the hydrazine absorption region serves as the ferromagnetic building block and the pristine Se–Nb–Se framework as the superconducting building block (Supplementary Fig. 8), representing the successful structural integration of SC and FM in a 2D nanomaterial.

In conclusion, we demonstrate the surface molecular adsorption of polar reductive hydrazine molecules to induce structural distortion in 2D NbSe_2_ nanosheets, which led to the successful expression of the magnetic moment of the Nb ions with ordered spin behaviour, thus representing freestanding case of the coexistence of SC and FM in one 2D nanomaterial. The surface distortion triggered by this molecular adsorption resulted in an elongated Nb–Se covalent bond, which weakened the covalent Nb–Se interaction and reduced the hybridization of Nb with Se atoms, thereby augmenting the unpaired electrons around the Nb ions and thus resulting in FM. Within this 2D superconducting framework, the induced ferromagnetic momentum coupled with conduction electrons to generate unique correlated effects of intrinsic negative MR and the Kondo effect. We anticipate that this strategy for surface-structural modification via polar molecular adsorption will provide versatile paths for the preparation of correlated systems with coexisting electronic phases.

## Methods

### Materials

Niobium powder (99.9%), selenium powder, iodine (analytical grade) and n-butyl-lithium (n-BuLi; 2.5 M in hexane) were purchased from SinopharmChemical Reagent Co. Ltd. Hydrazine hydrate (98%) was purchased from Aladdin Chemical Reagent Co. Ltd. Bulk NbSe_2_ was prepared following previously reported literature, which took advantage of the chemical vapour transport method with I_2_ as the transport agent. In detail, 1 mmol Nb, 2 mmol Se and 100 mg I_2_ powders were well mixed and sealed under vacuum (10^−5^ torr) inside a quartz tube, which is of 12 cm long and 0.8 cm inner diameter. The transport was achieved by establishing an 80°C gradient along the tube with the *T*_hot_=900 °C, and the mixture was heated for 36 h.The resultant was gathered and grounded for the following procedure.

### Preparation of NbSe_2_ nanosheets

In a typical procedure, Li_*x*_NbSe_2_ was synthesized via lithium insertion using 90 mg of NbSe_2_ powder in 1 ml of n-BuLi (2.5 M) diluted with 8 ml of hexane in a customized glass vacuum system reacted at 60 °C and was stirred for 12 h to obtain Li-inserted NbSe_2_. The resultant dispersions were filtered, washed with cyclohexane and anhydrous ethanol several times, and then vacuum-dried at 60 °C for the subsequent experimental procedures. Afterwards, 20 mg of the as-obtained Li_*x*_NbSe_2_ was dispersed in 20 ml of distilled water. Then, the solution was bubbled with nitrogen to expel any dissolved oxygen molecules to avoid oxidation. The Li_*x*_NbSe_2_-containing dispersion was ultrasonicated for 1 h. Afterwards, the resultant wine-red suspension was centrifuged at 2,000 r.p.m. for 20 min. The supernatant was then decanted into another container to remove unexfoliated flakes, and a high-quality dispersion of [NbSe_2_]^*n*−^nanosheets was thus prepared.

### Hydrazine treatment

The hydrazine treatment was similar to that applied to obtain reduced graphene oxide. First, 4 ml of a dispersion of highly exfoliated NbSe_2_ nanosheets was vacuum-filtered over a cellulose membrane with a 0.22-μm pore size to form a homogeneous thin film, the thickness of which could be tuned by modifying the filtration time, and the thickness of the film in the experiments was controlled to be 200 nm. Afterwards, the film was transferred on a Si/SiO_2_ substrate cleaned with ultraviolet oxygen radiation. The as-obtained film was subsequently treated via 5 min of exposure to hydrazine vapour generated at 70 °C. One subset of the hydrazine-vapour-treated samples was set aside for characterizations after the hydrazine vapour treatment. Other subset of assembled films was for the superconducting quantum interference device (SQUID) and physical property measurement system (PPMS) measurements.

### Characterization

X-ray diffraction was performed using a Philips X'Pert Pro Super diffractometer with Cu Kα radiation (*λ*=1.54178 Å). Transmission electron microscopy (TEM) images were obtained using a JEOL-2010 transmission electron microscope at an acceleration voltage of 200 kV. Field-emission scanning electron microscopy (FE-SEM) images were acquired using a JEOL JSM-6700F SEM. HR-TEM images were acquired using a JEOL 2010 microscope at an accelerating voltage of 200 kV. Scanning TEM (STEM) images and energy-dispersive X-ray spectra (EDS) were obtained using a JEM 2100F (field emission) transmission electron microscope equipped with an Oxford INCA X-sight EDS Si(Li) detector at an acceleration voltage of 200 kV. A Shimadzu IRPrestige-21 spectrometer with a standard 45° ZnSe ATR cell (Specac Ltd., Woodstock, GA) and a ZnSe grating polarizer (PIKE Technologies Inc., Madison, WI) was used to collect ATR-FTIR spectra. XAFS measurements at the Se *K*-edge (12 658 eV) were performed in fluorescence mode at the BL14W1 beamline of the Shanghai Synchrotron Radiation Facility (SSRF), China. The storage ring of SSRF was operated at 3.5 GeV with a maximum current of 210 mA. The magnetization was characterized using a SQUID (quantum design MPMS XL-7) magnetometer with a temperature range of 2–300 K and an applied field range of −3,000 to 3,000 Oe. The magnetic measurements were performed based on the assembled thin film of ultrathin NbSe_2_ nanosheets with and without hydrazine treatment, respectively. The background signal of Si/SiO_2_ substrate was treated by deducting the data of blank Si/SiO_2_ substrate. The magnetic-transporting property was measured via the four-probe technique using a Quantum Design physical property measurement system (PPMS-9) with the van der Pauw method. Gold contacts were evaporated on the sample through a stencil mask, and gold wires were embedded and connected to the gold contacts with silver paste to serve as the leads, which provided a strong electrical contact and consequently a small contact resistance. The transport measurement was performed on the assembled film with four-probe configuration. High-resolution Raman spectra were recorded at room temperature using a LABRAM-HR confocal laser micro Raman spectrometer at 750 K with a laser power of 0.5 mW.

### Density functional theory calculations

Electronic structure calculations and geometrical optimizations were performed using density functional theory as implemented in VASP 5.3 (refs 37, 38). The exchange-correlation energy was treated using the proton balance equation (PBE)-type GGA functional, which has been proven to be able to describe this system very well^30^. The ion–electron interaction was treated using the projector-augmented wave technique. A cutoff energy of 520 eV was adopted. For geometric optimization, both the lattice constants and the atomic positions were relaxed until the forces on the atoms were less than 0.02 eV Å^−1^ and the total change in energy was less than 1.0 × 10^−5^ eV. The Brillouin zone was sampled using k-points with a 0.02-Å^−1^ spacing in the Monkhorst–Pack scheme^39^ for geometry optimization, and for the DOS calculations, a denser grid with a 0.01-Å^−1^ spacing was used.

The adsorption energy calculation is performed using density functional theory as implemented in VASP 5.3. Exchange-correlation energy was treated using the PBE-type GGA functional. The ion–electron interaction was treated using the projector-augmented wave technique. A model of one N_2_H_4_ molecule adsorbed on 3 × 3 NbSe_2_ monolayer (9·NbSe_2_) or stacked bilayer (18·NbSe_2_) was adopted. The adsorbed structures were fully relaxed until the forces on atoms were less than 0.02 eV Å^−1^ and the total energy change was less than 1.0 × 10^−5^ eV. The adsorption energy is defined as 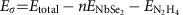, in which *E*_total_ is the total energy of N_2_H_4_ molecule-adsorbed NbSe_2_ monolayer or stacked bilayer, 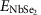
is total energy of a NbSe_2_ unit in pristine NbSe_2_ monolayer and 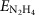
is the total energy of an isolated N_2_H_4_ molecule.

## Additional information

**How to cite this article:** Zhu, X. *et al*. Signature of coexistence of superconductivity and ferromagnetism in two-dimensional NbSe_2_ triggered by surface molecular adsorption. *Nat. Commun.* 7:11210 doi: 10.1038/ncomms11210 (2016).

## Supplementary Material

Supplementary Information Supplementary Figures 1-8, Supplementary Table 1, Supplementary Notes 1-3 and Supplementary References
