# Supplementary material for: Signature of coexistence of superconductivity and ferromagnetism in two-dimensional NbSe2 triggered by surface molecular adsorption
Source: Nat Commun. 2016 Apr 4;7:11210. doi: 10.1038/ncomms11210 (PMC4822027; doi:10.1038/ncomms11210)
Supplement: Supplementary Information — Supplementary Figures 1-8, Supplementary Table 1, Supplementary Notes 1-3 and Supplementary References [file ncomms11210-s1.pdf]

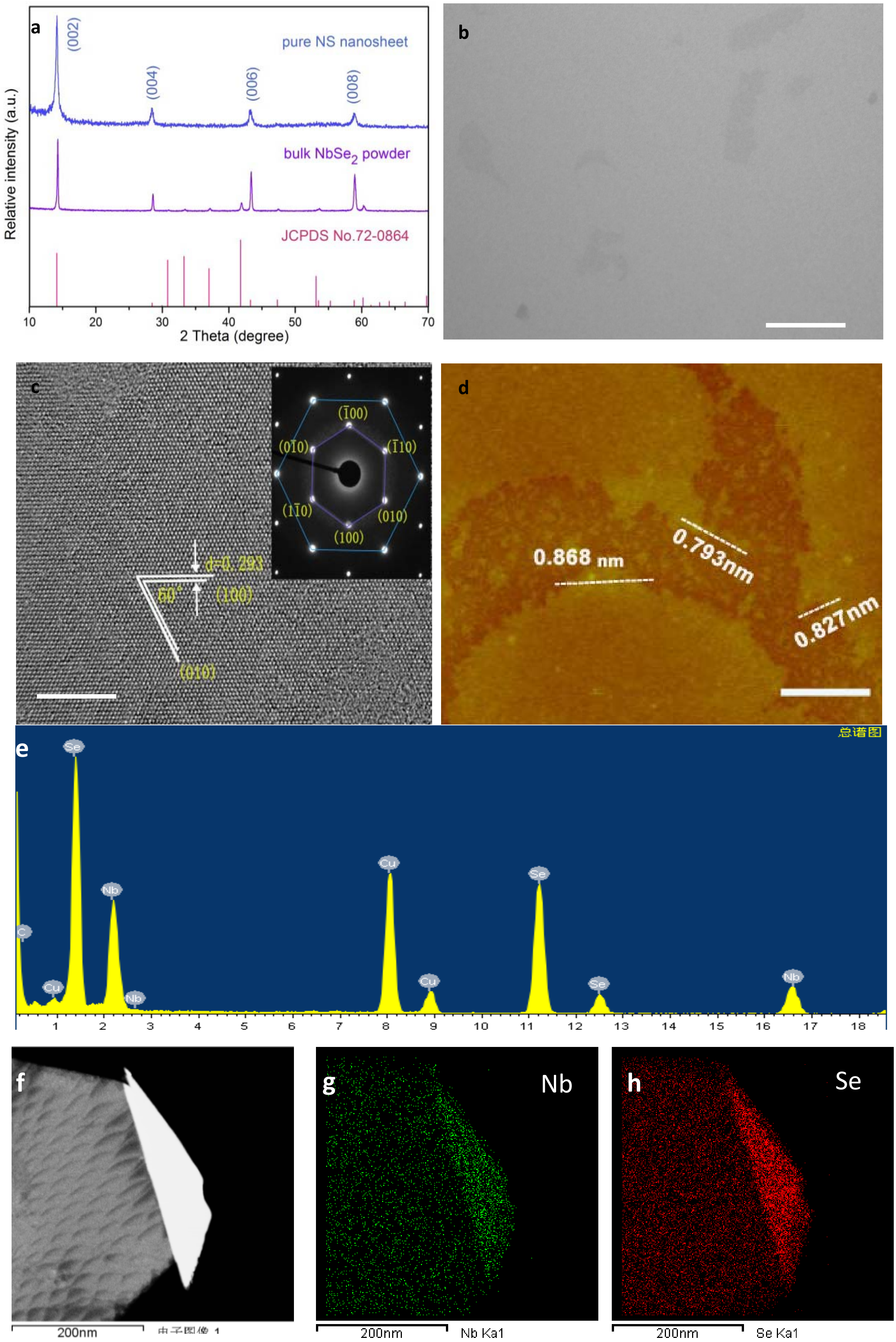
a
(002)
(004)
(006)
(008)
pure NS nanosheet
bulk NbSe2 powder
JCPDS No.72-0864
Relative intensity (a.u.)
10
20
30
40
50
60
70
2 Theta (degree)
b
c
(1̄00)
(01̄0)
(1̄10)
(11̄0)
(010)
(100)
d=0.293
60°
(100)
(010)
d
0.868 nm
0.793nm
0.827nm
e
总谱图
Se
Nb
C
Cu
Cu
Nb
Cu
Se
Se
Nb
1
2
3
4
5
6
7
8
9
10
11
12
13
14
15
16
17
18
f
200nm
g
Nb
200nm
Nb Ka1
h
Se
200nm
Se Ka1

Supplementary Figure 1|Systematical characterizations of $NbSe_2$ nanosheets. a, The XRD pattern of the as-assembled $NbSe_2$ film with highly c-orientation; **b**, TEM image of monolayer $NbSe_2$nanosheets; **c**, HR-TEM and SAED analyses of the obtained $NbSe_2$nanosheets; **d,** Tapping mode AFM image of the exfoliated $NbSe_2$nanosheets; Elemental ingredients analysis of $NbSe_2$ graphene analogue by HRTEM (**e-h**): **e**, EDX spectrum of a typical ultrathin nanosheet from (e), where the signals of Cu are generated from the Cu grids. (**f-h**), The typical HAADF-STEM (**f**) and (**g,h**) elemental mapping images of a single exfoliated $NbSe_2$ ultrathin nanosheet, where the elements of Nb (indicated by green color) and Se (indicated by red color) were homogenously spatial distributions in the entire nanosheet. The scale bars for **b**, **c** and **d** are 300 nm, 8 nm and 250 nm, respectively.

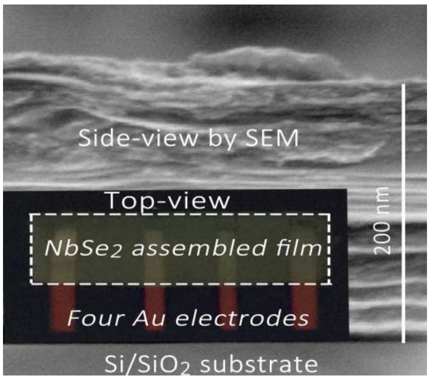

Supplementary Figure 2|Image of the assembled $NbSe_2$ film for the transport measurements. The SEM of cross-section of the assembled $NbSe_2$ film with four Au electrodes, inset for the top-view optical picture of the sample, and scale bar is 200 nm.

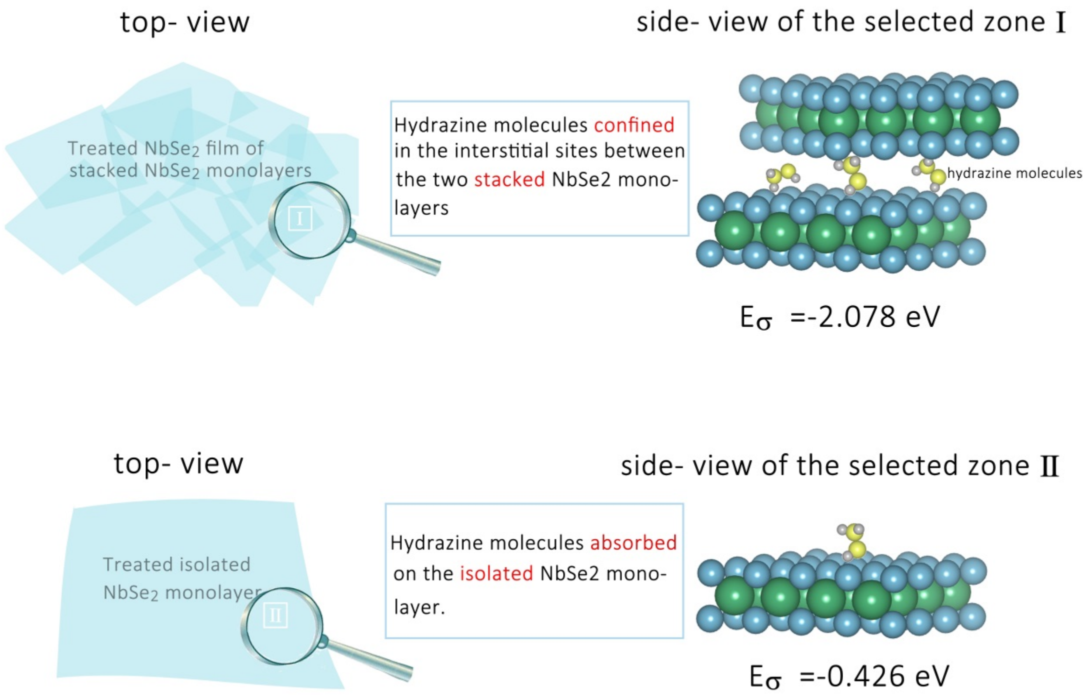

Supplementary Figure 3|Effective surface molecular adsorption of assembled $NbSe_2$ film.

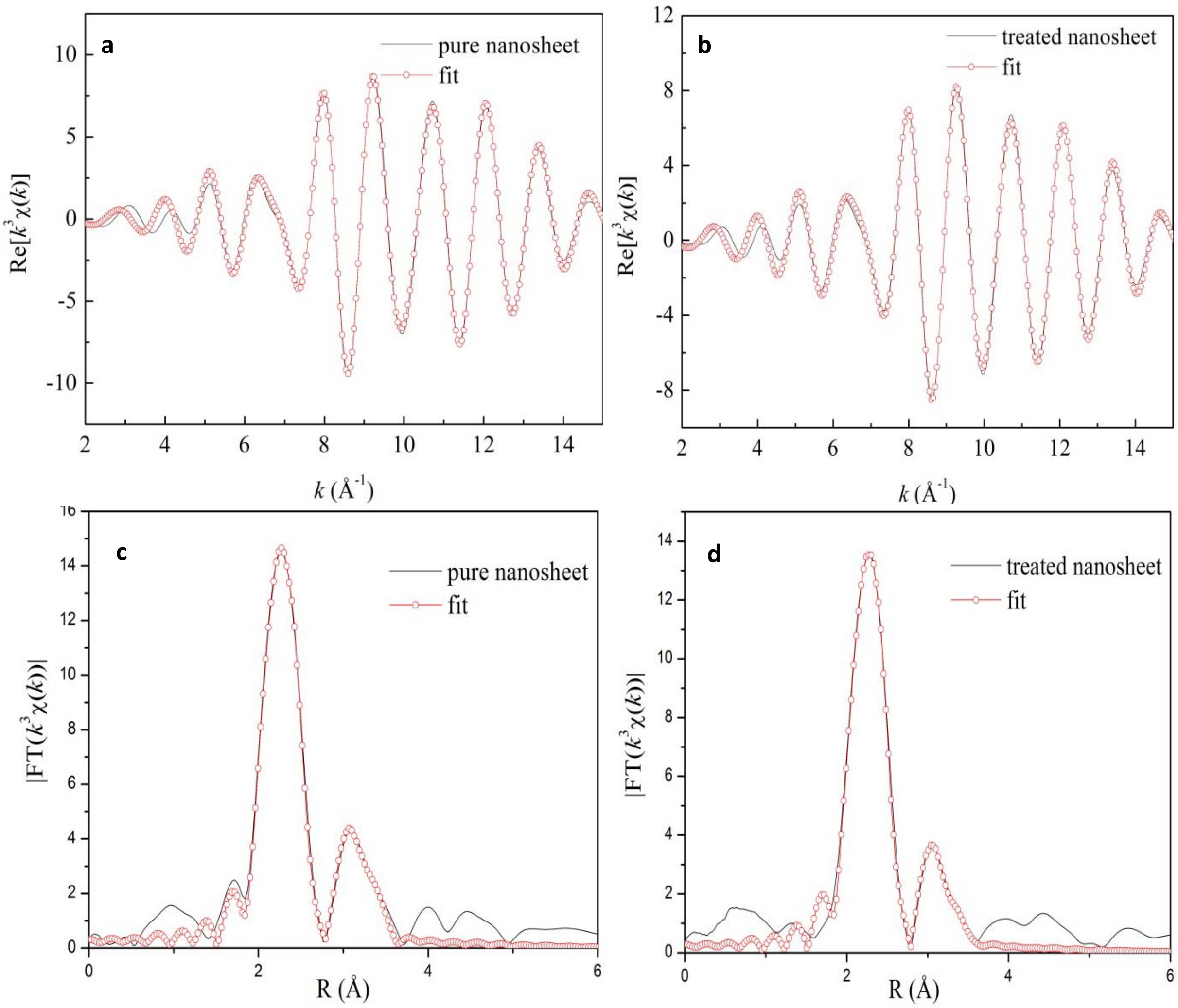

Supplementary Figure 4|Least-squares fittings for the Se *K*-edge data, and the obtained fitting curves and quantitative results. a and **b,** k_3-weighted Se *K*-edge EXAFS spectra; **c** and **d,** The Fourier-transformed magnitude of k_3-weighted Se *K*-edge and the corresponding fitting analysis for the pure $NbSe_2$nanosheets and the treated $NbSe_2$nanosheets.

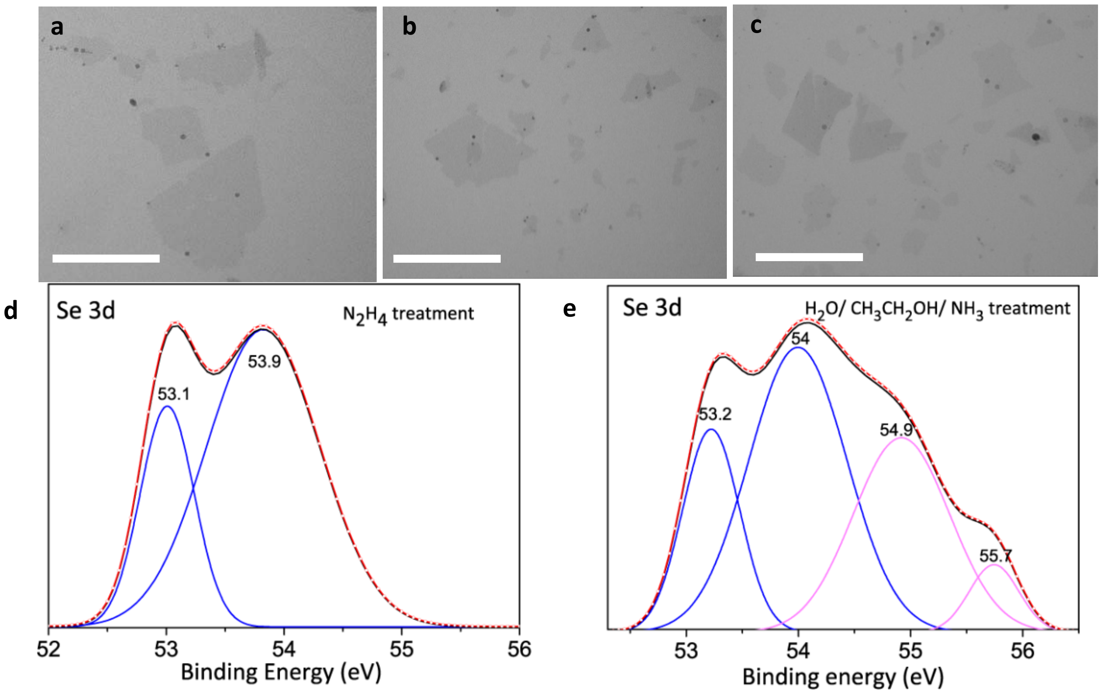

Supplementary Figure 5|Strong polar molecules with oxidative potential treated the $NbSe_2$ samples, bringing about the Se nanoparticles on $NbSe_2$ nanosheets. **a-c**, TEM images of the H_2O, CH_3CH_2OH and NH_3-treated $NbSe_2$ nanosheets, respectively; **d-e**, the Se 3d core spectra of hydrazine and H_2O / CH_3CH_2OH / NH_3-treated $NbSe_2$ nanosheets. The scale bars for **a**, **b** and **c** are 300 nm.

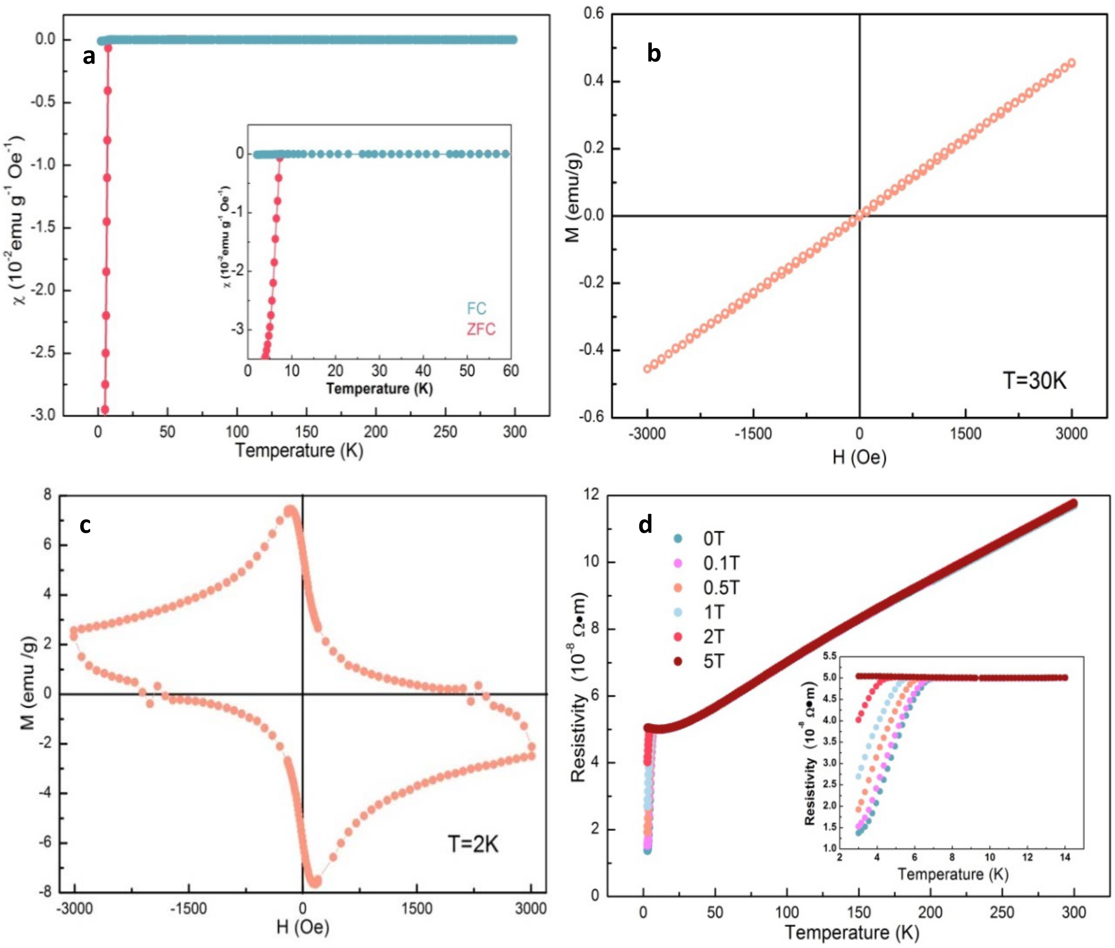

Supplementary Figure 6|The typical SC behavior delivered by pure $NbSe_2$nanosheets.a, The temperature dependence of magnetic susceptibility χ for the pure $NbSe_2$nanosheets under a magnetic field of 200 Oe with field-cooling (FC) and zero-field-cooling (ZFC) processes, where the inset displays magnified view of χ-T at low temperature. **b** and **c** Isothermal magnetizations (M-H curves) at 30K and 2K, respectively. **d**, The temperature dependence of resistivity under various magnetic fields, where the inset presents a magnified view of R-T at low temperature.

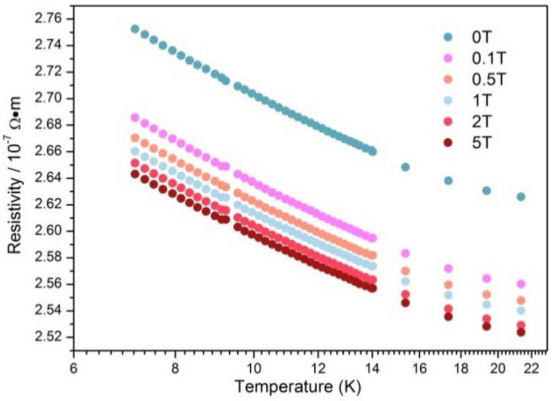

Supplementary Figure 7|The logarithmic temperature dependence of the resistivity performed on hydrazine-treated $NbSe_2$ nanosheets is consistent with Kondo scattering (ref 1) at a limited temperature range(7K<T<22K).

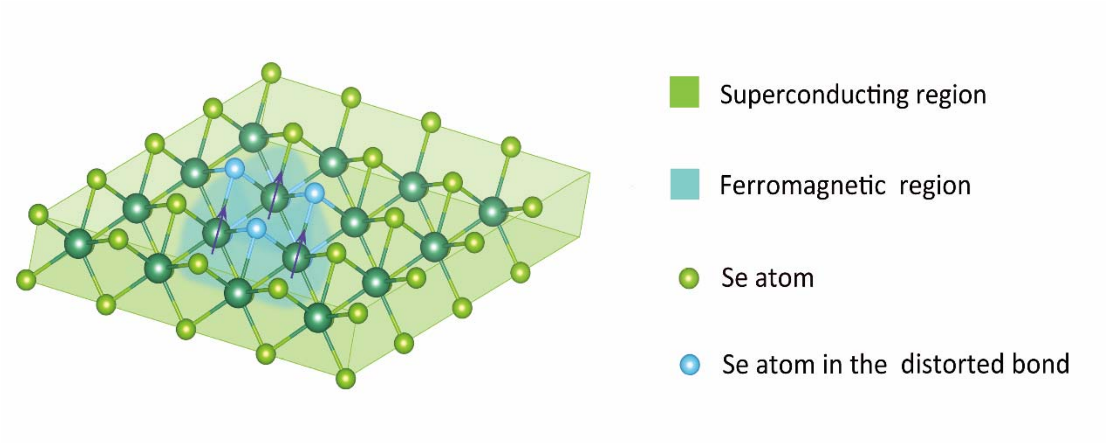

Supplementary Figure 8|Schematic illustration of the phase separation for the observation of superconductivity and ferromagnetism in the treated two-dimensional $NbSe_2$.

Supplementary Table 1| EXAFS curve-fitting results.

Sample	Path	N	R (Å)	$\sigma^2(10^{-3}Å^2)$	ΔE_0 (eV)
pure nanosheets	Se-Nb	3.0	2.58	4.6	1.7
	Se-Se	6.0	3.44	12.1	4.3
treated nanosheets	Se-Nb	3.0	2.60	4.9	1.6
	Se-Se	5.9	3.41	13.7	4.3

Supplementary Note 1

The systematical characterizations demonstrated that the $NbSe_2$ nanosheetswere successfully obtained. The XRD patterns provide direct phase information for the $NbSe_2$nanosheets. As can be seen in **Supplementary Figure 1a**, the pure $NbSe_2$ nanosheet exhibited the highly c-axis orientation where only (0 0 2l) peaks can be obviously observed, and further verified by their corresponding high-resolution TEM (HRTEM) image and the selected area electron diffraction (SAED) in **Supplementary Figure 1c**. As can be seen, the interplanar distance of 0.293 nm matched well with the plane distances of d_{100} and d_{010} of the facets of $NbSe_2$ (0.298 nm). The orientation angle values 60° of these two planes of (100) and (010) appeared in the SAED patterns and the lattice fringes in HRTEM image were in consistent with those calculated from hexagonal crystallographic parameters of 2H-$NbSe_2$. As illustrated in **Supplementary Figure 1d**, tapping mode AFM was also conducted to evaluate the thickness of the $NbSe_2$nanosheets. And the measured heights of the $NbSe_2$nanosheets regions were 0.793, 0.827 and 0.868 nm, corresponding to single layer, considering the c parameter of 2H-$NbSe_2$ is 1.254 nm. Energy-dispersive X-ray spectroscopy (EDXS) and elemental mapping

(**Supplementary Figure 1 e-h**) confirm the product has the chemical formula of $NbSe_2$, and no other obvious contaminants can be found in the as-obtained nanosheets.

Supplementary Note 2

To better understand the thermodynamic stability of the adsorbed hydrazine molecules in the interstitial, density function theory (DFT) calculations (details see the “Methods” section in the manuscript) are performed. As schematically shown in **Supplementary Figure 3**, hydrazine molecules confined in the interstitial sites possess the adsorption energy $\mathbf{E_{\sigma}}$(-2.078 eV), which is 4 times bigger than $\mathbf{E_{\sigma}}$(-0.426 eV) for molecules absorbed in the bare sites, indicating that hydrazine molecules prefer to be stabilized in the interstitial sites. Given the abundant interstitial sites in the assembled film, the film form is applied here as the suitable platform to achieve the effective surface-modification of $NbSe_2$.

Supplementary Note 3

To inspect whether polar molecules with oxidative potentials can be utilized to trigger the surface distortion, parallel experiments of the H_2O, $CH_3CH_2OH$and NH_3treatment on the pure $NbSe_2$nanosheets were also performed. As shown in **Supplementary Figure 5 a, b** and **c**, nanoparticles can clearly be seen on the $NbSe_2$ nanosheetsafter the H_2O, CH_3CH_2OH and NH_3 treatment, respectively. X-ray photoelectron spectra (XPS) were conducted to further demonstrate the oxidation of selenium by the polar moleculeswith relative lower reductive capability. As shown in **Supplementary Figure 5 d** and **e**, the Se 3d core spectra of H_2O / CH_3CH_2OH / NH_3-treated$NbSe_2$ nanosheets were prominently different from that of the hydrazine-treated $NbSe_2$nanosheets, which consist of quadruple peaks, suggesting the mixed selenium valence states, especially the additional doublet peak of Se^0 $3d_{3/2}$ (55.7 eV) and Se^0 $3d_{5/2}$ (54.9 eV) (ref 2).Therefore, strong polar molecules with relative lower reductive capability are inappropriate for the surface structural modulation which can’t maintain the integrity of original structure.

Supplementary References.

1. Chen, J.-H., Li, L., Cullen, W. G., Williams, E. D. & Fuhrer, M. S. Tunable Kondo effect in graphene with defects. Nat Phys 7, 535-538, (2011).
2. Babu, P. K. et al. Selenium Becomes Metallic in Ru−Se Fuel Cell Catalysts: An EC-NMR and XPS Investigation. Journal of the American Chemical Society 129, 15140-15141, doi:10.1021/ja077498q (2007).
